# Circadian Consequence of Socio-Sexual Interactions in Fruit Flies *Drosophila melanogaster*

**DOI:** 10.1371/journal.pone.0028336

**Published:** 2011-12-15

**Authors:** Shahnaz Rahman Lone, Vijay Kumar Sharma

**Affiliations:** Chronobiology Laboratory, Evolutionary and Organismal Biology Unit, Jawaharlal Nehru Centre for Advanced Scientific Research, Bangalore, Karnataka, India; Harvard University, United States of America

## Abstract

In fruit flies *Drosophila melanogaster*, courtship is an elaborate ritual comprising chasing, dancing and singing by males to lure females for mating. Courtship interactions peak in the night and heterosexual couples display enhanced nighttime activity. What we do not know is if such socio-sexual interactions (SSI) leave long-lasting after-effects on circadian clock(s). Here we report the results of our study aimed at examining the after-effects of SSI (as a result of co-habitation of males and females in groups) between males and females on their circadian locomotor activity rhythm. Males undergo reduction in the evening activity peak and lengthening of circadian period, while females show a decrease in overall activity. Such after-effects, at least in males, require functional circadian clocks during SSI as loss-of-function clock mutants and wild type flies interacting under continuous light (LL), do not display them. Interestingly, males with electrically silenced Pigment Dispersing Factor (PDF)-positive ventral lateral (LNv) clock neurons continue to show SSI mediated reduction in evening activity peak, suggesting that the LNv clock neurons are dispensable for SSI mediated after-effects on locomotor activity rhythm. Such after-effects in females may not be clock-dependent because clock manipulated females with prior exposure to males show decrease in overall activity, more or less similar to rhythmic wild type females. The expression of SSI mediated after-effects requires a functional olfactory system in males because males with compromised olfactory ability do not display them. These results suggest that SSI causes male-specific, long-lasting changes in the circadian clocks of *Drosophila*, which requires the presence of functional clocks and intact olfactory ability in males.

## Introduction

Although light/dark (LD) cycles are known to act as a primary zeitgeber for the circadian clocks of a wide variety of organisms, non-photic cues such as temperature, food and cyclic social interactions have been also shown to be equally effective [1], [2]. In many organisms cyclic presence and absence (PA) of other individuals from the same or other species have been shown to serve as time cue for the entrainment of circadian timing systems. For example in several mammals, PA cycles of conspecifics are able to entrain circadian activity rhythm [3]–[10]. In the bat species *Hipposideros speoris*, PA cycles of free-living conspecifics entrain circadian activity rhythm of individuals maintained in captivity inside a cave [5], while activity rhythm of a different species of an emballonurid bat (*Taphozous nudiventris kachhensis*), also kept captive in the same cave continues to free-run, suggesting that, in bats, social communication of temporal information is species-specific [7]. In mice, PA cycles of the mother entrain circadian activity rhythm of pups [6] for at least 23–26 days of post-natal development [8]. Similarly, in the Siberian hamster *Phodopus sungoru*, PA cycles of foster mothers entrain rhythms of pups that were previously entrained 12 hr out-of-phase with their biological mothers [9]. In European rabbits *Oryctolagus cuniculus*, nursing by mothers just for few minutes every day entrains oscillation of clock gene expression in the hypothalamus [10]. The results of the above studies suggest that cyclic social cues can serve as zeitgeber for the circadian clocks of a variety of mammalian species.

In fruit flies *Drosophila melanogaster*, lesser known for its social organization, social cues have been reported to influence circadian clocks [11]–[15]. In an elegant study Levine et al [11] showed that flies maintained in groups show greater phase synchrony compared to those maintained solitarily. The time of social interactions is critical in maintaining such synchrony and olfaction seems to be critical in the communication of social signals [11]. In a similar and more recent study it was shown that presence of *per^0^* flies in a group of wild type *CS* individuals altered the behaviors and transcript levels of core clock genes in the head and oenocytes of the wild type hosts [13]. These studies suggest that fruit flies can convey temporal information by socially interacting with each other. However, social cycles were unable to entrain the circadian clocks of *D. melanogaster*, although they had significant impact on the phase of circadian locomotor activity rhythm [14], [15].

Courtship in *Drosophila* comprise of a series of well defined behaviors such as chasing, avoiding, dancing, rejection and copulation, all of which involve intense locomotor activity by both males and females [16]. Circadian clocks are known to regulate inter-pulse song intervals during courtship [16], [17]. Not just the courtship song but the act of mating itself is rhythmic at least in some species of fruit flies - *D. melanogaster*, *D. yakuba*, *D. kikkawai* and *Dacus oleae*, with a peak just before lights-on [18], [19], and is believed to be primarily dictated by females [20]. In an elegant study Fujii et al [12] studied “nocturnal sex drive” in *D. melanogaster* and found that while solitary, sex-starved, virgin males and females are day active and night inactive, heterosexual couples are active during night and most part of the day, and were least active only for a brief period in the evening. What we do not know is whether such changes in the activity rhythm is temporary and would disappear as soon as males and females are separated from each other. To the best of our knowledge long-term consequence of socio-sexual interactions (SSI) between males and females on circadian clocks has never been reported.

Here we report the results of our study aimed at examining the consequence of SSI on the circadian locomotor activity rhythm of fruit flies *D. melanogaster*. We first maintained males and females in same or mixed sex groups (comprising of ∼30 individuals in each group) under 12∶12 hr light/dark (LD) cycles, for different days, and then separated them to individually record their locomotor activity behavior under LD, or constant dark (DD) conditions. Age matched flies maintained in male-only or female-only groups of 30 individuals per group served as controls. By maintaining flies in mixed sex groups for different durations we wanted to find out if changes in circadian activity rhythm occur, and how many days of SSI are needed to make an appreciable impact. To study if circadian clocks play any role in the regulation of such after-effects, we used loss-of-function mutants for core clock genes, or allowed wild type flies to interact under constant light (LL, wherein wild type flies are known to become arrhythmic [21]), or flies with electrically silenced Pigment Dispersing Factor (PDF)-positive ventral lateral (LNv) clock neurons. Further, we studied the role of olfaction in the expression of SSI mediated after-effects on circadian activity rhythm. We found evidence for circadian consequences of SSI in males, which was absent in flies lacking functional circadian clocks or olfactory ability.

## Materials and Methods

### Fly strains

To study if SSI causes any long-lasting after-effect on the circadian activity rhythm we used wild type *Canton S* (*CS*) flies. To examine the role of circadian clocks we took flies with loss-of-function mutation for core clock genes such as *period* (*per^0^*) [22] and *cycle* (*cyc^0^*) [23], or flies with electrically silenced PDF-positive LNv neurons (*pdfGAL4/UASdORKC1*) [24]. To elucidate the role of olfaction we used flies with loss-of-function mutation in a widely expressed olfactory receptor *Or83b* (*Or83b^0^*) [25] or flies whose *Or83b* receptor neurons were ablated (*Or83b^−^*; *Or83bGAL4/UASrpr*). To determine if circadian clocks are required during SSI to express its after-effects later on we co-housed wild type males and females under LL only for the period of SSI and then monitored their locomotor activity rhythm individually under LD or DD conditions.

### Experimental protocol for the study of after-effects of SSI

Eggs from different *Drosophila* strains were collected in glass vials (95 mm length ×10 mm diameter) and maintained under 12∶12 hr LD cycles with light of intensity ∼100 lux during the day and red light of wavelength >650 nm during the night. Only virgin flies were used in the experiments to avoid any confounding effect of previous mating and habituation.

#### Experiment-1A, B

The general experimental protocol for the experiments is illustrated in detail in Figure S1. Freshly emerged virgin *CS* males and females were collected and maintained under 12∶12 hr LD cycles (with ∼100 lux light intensity during light phase) for 4 days as same-sex groups of 30 individuals per vial. The above step is common to all our experiments. After 4 days, flies were segregated into five sets (experiment 1A), of which the first was maintained as same sex groups (males or females) of 30 flies per vial (henceforth will be referred as flies with 0 days of SSI, or simply control flies) until age day10. The remaining four comprised of males and females that were initially housed as same-sex groups of 30 flies but were mixed in 1∶1 ratio (with 15 ♂+15 ♀ per vial, henceforth will be referred as SSI flies) on different days. This enabled SSI to take place at different stages for each of the four groups. Males and females of one set were mixed on the 5^th^ day and kept together for next five days (5 days of SSI), of the next set were mixed on the 6^th^ day and kept together for next four days (4 days of SSI), of another set were mixed on the 7^th^ day and kept together for next three days (3 days of SSI) and of the last set were mixed on the 8^th^ day and then kept together for next two days (2 days of SSI), such that SSI in all the interacting groups ended on the same day. Thus, SSI was initiated on different days in the four sets of flies to ensure that the age of flies on the day of conclusion of SSI is the same. This was done to avoid any confounding effect of age on SSI. Flies (SSI and control) were transferred into fresh food vials every day until the termination of SSI. On completion of SSI (age day 10 for all SSI groups), males from both SSI and control sets were introduced individually into glass tubes to monitor their locomotor activity behavior under LD cycles for at least 5 days.

To examine if SSI mediated after-effects on circadian activity rhythm, if any, are stable and reproducible, we carried out a separate but very similar experiment (experiment 1B, Figure S1) where three SSI sets comprised males and females co-housed in 1∶1 ratio (with 15 ♂+15 ♀ per vial) for 5 days, 8 days, or 10 days. Here controls (0 days SSI) remained in same-sex groups till age day14. Males and females of one SSI set were mixed on the 5^th^ day and kept together for next ten days (10 days of SSI), the second set were mixed on the 7^th^ day and kept together for next eight days (8 days of SSI) and the third set were mixed on the 10^th^ day and kept together for next five days (5 days of SSI). Thus SSI was initiated on different days in the three SSI sets of flies to ensure that age of flies on the day of conclusion of SSI is the same. This was done to avoid any confounding effect of age on SSI. Flies (SSI and control) were transferred into fresh food vials every day until the termination of SSI (age day 14). On completion of SSI (age day 14), males from SSI and control sets were introduced individually into glass tubes to monitor their locomotor activity behavior under LD cycles. The protocol of this experiment was slightly different from the previous experiment and we have presented the results in Fig. S2.

To study the circadian period under DD, we used 4 day old *CS* virgin males and females and mixed them in 1∶1 ratio to form groups of 30 flies per vial. These flies were maintained for 5 days under LD as mixed-sex groups and thereafter separated and introduced into DD as isolates for locomotor activity recording. For controls, 4 day old virgin males were maintained for 5 days under LD as same sex groups of 30 flies per vial, and thereafter introduced into DD for locomotor activity recording. Individual flies used in this assay were age-matched to avoid any confounding effect of age on the circadian locomotor activity rhythm.

In a separate experiment we examined whether change in circadian period, if any, is stable and could be sustained if SSI is followed by social interactions among members of the same sex before testing them individually in DD. For this 4 day old males were first kept together with females in 1∶1 ratio in groups (with 30 individuals in each vial) under LD cycles for 5 days and then segregated into male only groups of 30 males per vial for 8 days before being transferred to DD in individual glass vials for assaying locomotor activity behavior. Age matched control males were maintained for 13 days in a similar manner but in male only groups of 30 individuals per vial and thereafter segregated and introduced individually into activity tubes for locomotor activity assay.

#### Experiment-2

To study circadian consequence of SSI on locomotor activity rhythm in females, flies were subjected to exactly the same treatments as in experiment 1 except that in this case duration of SSI was 5 days and locomotor activity behavior of females was recorded. We took 4 day old virgin males and females, mixed them in 1∶1 ratio (with 30 individuals in each vial) and maintained them together in groups under LD cycles for a period of 5 days. Flies kept in female only groups (comprising of 30 virgin females per vial) under LD cycles for a period of 5 days served as controls. After 5 days, both SSI and control females were transferred individually into glass tubes for recording locomotor activity behavior under LD. Females did lay eggs prior to and after separation from their groups; however, they were transferred into fresh food vials every day to minimize any confounding effect of presence of eggs or larvae on the locomotor activity behavior.

#### Experiment-3

In this experiment, flies were subjected to exactly the same treatments as in experiments 1, except that in this case the duration of SSI was 5 days and loss of function clock mutants *per^0^* and *cyc^0^* were used. We mixed 4 day old virgin males and females in 1∶1 ratio, and maintained them together in groups (with 30 individuals in each vial) for a period of 5 days under LD cycles. Flies kept in male-only or female-only groups under LD cycles for a similar period of time served as controls. After 5 days, SSI and control flies were separated and their locomotor activity behavior was monitored individually under LD cycles.

#### Experiment-4

To study if functional clocks are necessary during SSI for its after-effect to be seen later on, we allowed SSI to take place under LL (constant light of intensity ∼100 lux). For this, 4 day old wild type *CS* males and females were mixed in 1∶1 ratio (with 30 individuals in each vial) and introduced into LL for 5 days. Flies in male only or female only groups, kept under LL for a period of 5 days, served as controls. After 5 days, both SSI and control flies were separated and their locomotor activity behavior was monitored individually under LD or DD conditions.

#### Experiment-5

To study the role of LNv clock neurons in SSI mediated after-effect on circadian activity rhythm we took flies in which these neurons were electrically silenced by ectopically expressing potassium channels (*pdfGAL4/UASdORKC1*). In this experiment, flies were subjected to exactly the same treatments as in experiment 1, except that the duration of SSI was 5 days and LNv-silenced flies were used. We took 4 day old virgin males and females, mixed them in 1∶1 ratio in groups of 30 flies per vial, and maintained them for a period of 5 days under LD cycles. Flies kept for a period of 5 days under LD cycles, in male only and female only groups of 30 flies per vial, served as controls. After 5 days, both SSI and control flies were separated and their locomotor activity behavior was monitored individually under LD.

#### Experiment-6

To elucidate the role of olfaction in the regulation of SSI mediated after-effect on the locomotor activity rhythm we used flies with loss of function mutation in a widely expressed olfactory receptor *Or83b* (*Or83b^0^*) or flies whose *Or83b* receptor neurons were ablated by the expression of the apoptotic gene *reaper* (*Or83bGAL4/UASrpr*, henceforth will be referred as *Or83b^−^* flies). We took 4 day old virgin males and females, mixed them in 1∶1 ratio, and maintained for a period of 5 days under LD cycles, in groups of 30 individuals per vial. Flies in male only and female only groups, kept under LD cycles for a period of 5 days, served as controls. After 5 days, both SSI and control flies were separated and their locomotor activity behavior was monitored individually under LD.

To study the consequence of SSI on circadian period, we took 4 day old *Or83b^0^* virgin males and females, mixed in 1∶1 ratio, and maintained them together as groups for 5 days under LD cycles. Four day old flies, kept for a period of 5 days under LD cycles, in male only groups of 30 flies per vial, served as controls. After 5 days, SSI and control flies were separated and their locomotor activity behavior was monitored individually under DD.

### Recording locomotor activity behavior

For recording locomotor activity behavior, flies were placed individually in activity tubes (5 mm×65 mm) with corn food at one end and cotton plug at the other. The tubes were placed in Drosophila Activity Monitors (DAM), Trikinetics, USA. Food in the activity tubes of females (SSI and control) was changed once every day at random hours to avoid any larval movement and physiological effects of eggs in the food medium. Locomotor activity rhythm of males was recorded under both 12∶12 hr LD and under DD conditions while females were recorded only under LD. Recording the activity of mated females in DD was found to be not feasible because females laid many eggs and larvae hatched out which interfered with the locomotor activity recording of adult females. While this was tackled under LD by changing food regularly at random phases of the light duration, such steps when performed under DD were found to cause phase shifts in the locomotor activity rhythm and very difficult to carry out.

### Plotting and analyzing locomotor activity data

The activity data was analyzed using Lomb Scargle Periodogram of CLOCKLAB, Actimetrics, USA. In accordance with convention, lights-on was taken as ZT00, while lights-off was ZT12. Activity data was summed in 1 hr bins such that activity in the ZT01 bin would be a part of daytime activity and that in ZT13 bin of nighttime activity. Cumulative daytime and nighttime activity was obtained by summing up daytime or nighttime activity counts, respectively. Anticipation to lights-on or lights-off was calculated in terms of anticipation index (AI) using the formulae proposed by Stoleru and coworkers [26]. The AI for lights-on or lights-off was calculated by evaluating build-up of activity in three 1 hr bins prior to light-switches (lights-on or lights-off) and one 1 hr bin after. The activity in the bin immediately before the light switches was multiplied by the difference in activity during that bin and the one preceding it. Subsequently this value was multiplied by the difference in activity between the third and the second bins preceding light-switches. The resultant value was divided by activity of the bin immediately after light-switches.

In every experiment, data from individual flies were taken as replicate for analysis of variance (ANOVA). For the two-way ANOVA on activity data of experiment 1A, number of days of SSI (2, 3, 4 and 5 days) and time points (every hour between ZT01 and ZT24) were treated as fixed factors. Two-way ANOVA was also performed on the data obtained from a separate experiment (experiment 1B) with number of days of SSI (5, 8 and 10 days) and time points as fixed factors. For the experiment 2, two-way ANOVA was performed on the data obtained from females with social status (with or without SSI) and time points as fixed factors. For both the above ANOVAs, flies with 0 days of SSI were considered as control flies. For ANOVA on data from other experiments (experiments 3–6), sex (male or female), social status (with or without SSI) and time points were taken as fixed factors. Following the ANOVA, all those main effects and interactions that were significant at *p*<0.05 were subjected to post-hoc multiple comparisons using Tukey's test. Three-way ANOVA on cumulative activity data was performed by taking sex, SSI and phase (day or night) as fixed factors. Statistical analysis of data was implemented using STATISTICA^TM^ for Windows Release 5.0 B [27].

## Results

### Experiment-1: SSI reduces evening activity peak and lengthens clock period in males

**Figure 1:**
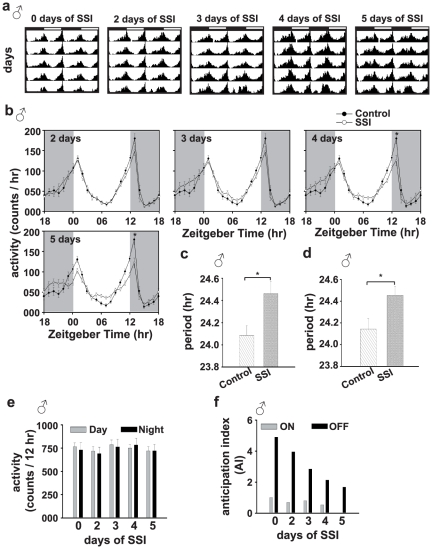
Socio-sexual interactions (SSI) reduce evening activity peak and lengthens clock period in males. (a) Average actograms of males following 0 (control), 2, 3, 4, or 5 days of SSI. (b) Activity profiles of males following 0–5 days of SSI. Zeitgeber Time (in hours) is plotted along x-axis, while activity (in counts in one hour bins averaged across five days) is plotted along y-axis. (c) The circadian period of locomotor activity rhythm of males following 5 days of SSI is lengthened compared to control males (*p*<0.01). (d) Circadian period of males kept in same sex groups (under light/dark – LD cycles) following 5 days of SSI for 8 days. The circadian period of SSI males is significantly lengthened compared to control males (*p*<0.02). (e) Cumulative daytime and nighttime activity of males subjected to 0, 2, 3, 4, or 5 days of SSI. The number of days of SSI is plotted along x-axis and daytime or nighttime activity (in counts/12 hr average across five successive days) along y-axis. (f) Anticipation to lights-on and lights-off as measured by Anticipation Index (AI) of male flies subject to 0, 2, 3, 4, or 5 days of SSI. The shaded area indicates night and the empty area day of laboratory LD cycles. The error bars are standard error around the mean (SEM). Asterisks indicate significant differences with *p* values<0.05.

The evening activity peak (activity in 1 hr bin between ZT12 and ZT13, where ZT00 is taken as lights-on and ZT12 as lights-off) of males exposed to females for 4 days or 5 days is significantly reduced compared to control males, while at all other time points their activity does not differ from controls (Fig. 1a, b). This was further confirmed in another set of experiments where males were exposed to females for 5 days or more (Fig. S2). The SSI males show a lengthening of circadian period compared to control males (Fig. 1c, d).

Two-way ANOVA on the activity data (experiment 1A) showed a statistically significant effect of number of days of SSI (*F*_4,3720_ = 2.90, *p*<0.02), time point (*F*_23,3720_ = 108.00, *p*<0.0001) and number of days of SSI×time point interaction (*F*_92,3720_ = 1.90, *p*<0.0001). Post hoc multiple comparisons using Tukey's test revealed that evening activity peak of 4, or 5 day SSI males is significantly lower than control males (*p*<0.0001; Fig. 1b). Although there is a change in the morning activity peak of males exposed to SSI for 5 days (Fig. 1b), it did not reach statistical levels of significance (*p*>0.05). The evening activity peak shows signs of reduction in males exposed to as few as 2 days of SSI but it takes a minimum of 4 days of SSI for the differences to reach statistically significant levels (*p*<0.0005 for 4 days and *p*<0.0002 for 5 days; Fig. 1b). Two-way ANOVA on cumulative activity data showed that effect of number of days of SSI (*F_4_*_,310_ = 0.47, *p* = 0.75), phase (day/night) (*F*_1,310_ = 0.06, *p* = 0.80) and SSI×phase interaction is statistically not significant (*F*_4,310_ = 0.10, *p* = 0.98; Fig. 1e). There is a gradual decrease in the lights-off AI with increasing number of days of SSI, while there is no measurable change in lights-on AI (Fig. 1f).

SSI mediated decrease in evening activity peak is further corroborated in a separate set of experiments (experiment 1B) wherein we subjected flies to 5, 8, or 10 days of SSI (Fig. S2). Two-way ANOVA on the activity data revealed a statistically significant effect of number of days of SSI (*F*_3,2688_ = 36.14, *p*<0.0001), time point (*F*_23,2688_ = 179.10, *p*<0.0001) and number of days of SSI×time point interaction (*F*_69,2688_ = 1.84, *p*<0.0001). Consistent with the results of our previous experiment, we find that the evening activity peak of males exposed to SSI for 5 (ZT12 - *p*<0.002 and ZT13 - *p*<0.0001), or 8 (ZT12 - *p*<0.0005 and ZT13 - *p*<0.0001), or 10 days (ZT13 - *p*<0.0001) is significantly reduced compared to control males, while at all other time points activity of SSI and control males does not differ (*p*>0.05; Fig. S2).

To estimate the circadian period of activity rhythm under DD, we recorded locomotor activity of males exposed to 5 days of SSI (Fig. 1c). ANOVA on the circadian period data showed a significant effect of SSI (*F*_1,112_ = 7.11, *p*<0.01). Post hoc comparison using Tukey's test revealed that circadian period of SSI males is significantly longer than control males (*p*<0.01; Fig. 1c). Further, to examine if such period lengthening is stable, and persists over an extended period of time long after SSI is over, we subjected males first to 5 days of SSI, segregated and maintained them in male-only groups for 8 days before recording their locomotor activity behavior under DD. Males with prior SSI exposure show a significant lengthening in circadian period compared to control males (Fig. 1d). ANOVA on the circadian period data showed significant effect of SSI (*F*_1,49_ = 5.70, *p*<0.02). Post hoc comparison using Tukey's test revealed that circadian period is significantly lengthened in SSI males compared to control males (*p*<0.02) (Fig. 1d). These results thus suggest that circadian period of males change as a consequence of SSI, and that such period changes are fairly stable over an extended period of time, long after SSI are terminated.

Thus, our first set of experiments suggests that SSI between males and females cause reduction in the evening activity peak and lengthening of circadian period in males, which persists long after they are separated from females. Since 5 days of SSI was sufficient to elicit a significant level of after-effects we decided to use 5 days of SSI for further experiments.

### Experiment-2: SSI makes females less active

**Figure 2:**
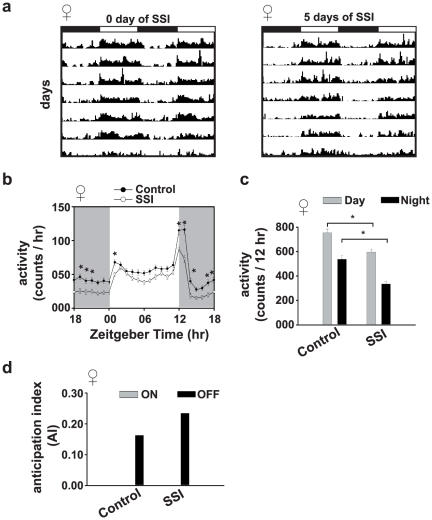
Socio-sexual interactions (SSI) cause reduction in overall activity in females. (a) Average actograms of females following 0 (control), or 5 days of SSI. (b) Activity profiles of females following 0, or 5 days of SSI. The activity of females is significantly reduced during morning and evening activity peaks and during most nighttime phases (*p*<0.05) in SSI females relative to control females. (c) Cumulative daytime and nighttime activity of females subjected to 0, or 5 days of SSI. (d) Anticipation to lights-on and lights-off measured as Anticipation Index (AI) of female flies subject to 0, or 5 days of SSI. All other details same as in Fig. 1.

The activity of SSI females is reduced compared to control females for most phases of the day (Fig. 2a, b). Two-way ANOVA showed a significant effect of SSI (*F*_1,3024_ = 360, *p*<0.0001), time point (*F*_23,3024_ = 102, *p*<0.0001) and SSI×time point interaction (*F*_23,3024_ = 4.5, *p*<0.0001). Post hoc multiple comparisons using Tukey's test revealed that SSI females are less active than controls during most subjective night phases (*p*<0.01). Two-way ANOVA on cumulative daytime and nighttime data showed a significant effect of SSI (*F*_1,240_ = 48.10, *p*<0.0001) and phase (day/night) (*F*_1,240_ = 97.2, *p*<0.0001), however, effect of SSI×phase interaction is statistically not significant (*F*_1,240_ = 2.95, *p* = 0.09). Post hoc comparisons revealed that cumulative daytime (*p*<0.005) and nighttime (*p*<0.0001) activity of SSI females is significantly reduced (Fig. 2c), suggesting that SSI makes females less active. The lights-off AI is increased in SSI females in comparison to control females, while there is no change in lights-on AI (Fig. 2d).

### Experiment-3: SSI mediated reduction in evening activity peak requires the presence of functional circadian clocks

**Figure 3:**
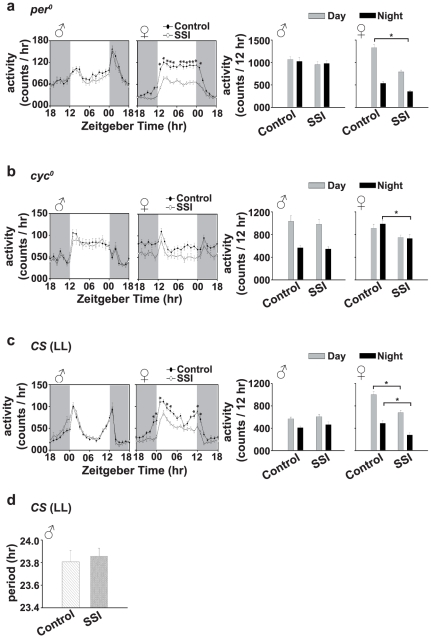
Clock mutant males do not display socio-sexual interaction (SSI) mediated after-effects. Activity profiles of (a, left panels) *per^0^* and (b, left panels) *cyc^0^* males and females after 0 (controls), or 5 days of SSI. In *per^0^* and *cyc^0^* flies, activity profiles of SSI and control males do not differ (a, b, left panels). Activity of *per^0^* females is reduced during most daytime phases (*p*<0.05), whereas at all other time points activity is similar to controls. In *per^0^* and *cyc^0^* flies, cumulative daytime and nighttime activity of SSI males (a, b, right panels) is similar to control males. In SSI females, cumulative daytime activity of *per^0^* flies and nighttime activity of *cyc^0^* females is reduced (*p*<0.05) in comparison to controls, while daytime activity of *cyc^0^* and nighttime activity of *per^0^* flies is similar to controls (*p*>0.05). (c, left panels) The figure shows that circadian phenotype of SSI males interacting under constant light (LL) is similar to controls. In females, activity is reduced at most time points tested (*p*<0.05) (c-left panels). (c, right panels) Cumulative daytime and nighttime activity of SSI males interacting with females in LL is similar to controls, while in SSI females it is reduced (*p*<0.05). (d) The circadian period of SSI males interacting with females under LL is similar to controls. All other details same as in Fig. 1.

Unlike the effects of SSI in wild type flies, we find that when *per^0^* and *cyc^0^* flies were exposed to SSI, the evening activity peak of SSI males does not differ from control males (Fig. 3a, b, left panels), suggesting a role of circadian clocks in SSI mediated reduction in evening activity peak.

Three-way ANOVA on the activity data of *per^0^* flies revealed a statistically significant effect of sex (*F*_1,2976_ = 275.65, *p*<0.0001), SSI (*F*_1,2976_ = 208.90, *p*<0.0001), time point (*F*_23,2976_ = 47.61, *p*<0.0001), sex×SSI (*F*_1,2976_ = 84.35, *p*<0.0001), sex×time point (*F*_23,2976_ = 47.61, *p*<0.0001), SSI×time point (*F*_23,2976_ = 3.15, *p*<0.0001) and sex×SSI×time point interaction (*F*_23,2976_ = 1.90, *p*<0.006). Post hoc multiple comparisons using Tukey's test revealed that activity of SSI males does not differ from control males at any time point tested (*p*>0.05), while in SSI females it is significantly reduced at almost all time points during the day (*p*<0.02; Fig. 3a, left panels). Three-way ANOVA on the cumulative daytime and nighttime activity data showed a significant effect of sex (*F*_1,248_ = 37.13, *p*<0.0001), SSI (*F*_1,248_ = 28.14, *p*<0.0001), phase (day/night) (*F*_1,248_ = 56.68, *p*<0.0001), sex×SSI (*F*_1,248_ = 11.37, *p*<0.0008), sex×phase (*F*_1,248_ = 53.53, *p*<0.0001) and SSI×phase (*F*_1,248_ = 6.56, *p*<0.01), however, effect of sex×SSI×phase interaction was statistically not significant (*F*_1,248_ = 3.03, *p* = 0.08). Post hoc multiple comparisons using Tukey's test revealed that cumulative daytime and nighttime activity of SSI and control males does not differ statistically (*p*>0.05), while daytime activity of SSI females is significantly reduced (*p*<0.0001) and nighttime activity is similar to control females (*p* = 0.36; Fig. 3a, right panels), suggesting that SSI makes *per^0^* females less active.

In *cyc^0^* flies, activity in males does not differ at any time point between SSI and control males, while in females activity is consistently lower at almost all time points of the day (Fig. 3b, left panels). Three-way ANOVA on the activity data of *cyc^0^* flies showed a statistically significant effect of sex (*F*_1,2976_ = 11.43, *p*<0.0007), SSI (*F*_1,2976_ = 128.03, *p*<0.0001), time point (*F*_23,2976_ = 9.75, *p*<0.0001), sex×SSI (*F*_1,2976_ = 42.83, *p*<0.0001), sex×time point (*F*_23,2976_ = 9.35, *p*<0.0001), however, SSI×time point (*F*_23,2976_ = 0.58, *p* = 0.94) and sex×SSI×time point interactions are statistically not significant (*F*_23,2976_ = 0.32, *p* = 1.0). Post hoc multiple comparisons using Tukey's test revealed that activity of SSI males and females does not differ from control males at any time point tested (*p*>0.05; Fig. 3b, left panels). Three-way ANOVA on the cumulative daytime and nighttime data showed a significant effect of sex (*F*_1,248_ = 6.24, *p*<0.01), SSI (*F*_1,248_ = 6.24, *p*<0.01), sex×SSI (*F*_1,248_ = 12.77, *p*<0.0004), sex×phase (*F*_1,248_ = 4.63, *p*<0.03) and SSI×phase interaction (*F*_1,248_ = 4.63, *p*<0.03), however, the effect of phase (day/night) (*F*_1,248_ = 0.98, *p* = 0.97) and sex×SSI×phase interaction are statistically not significant (*F*_1,248_ = 2.95, *p* = 0.09). Post hoc multiple comparisons using Tukey's test revealed that daytime activity of *cyc^0^* SSI males and females and nighttime activity of males does not differ from controls (*p*>0.05), while nighttime activity of females is significantly lower than control females (*p*<0.02; Fig. 3b, right panels). These results suggest that functional clocks in males are required for SSI mediated reduction in evening activity peak, while absence of clocks in females does not seem to matter for SSI mediated reduction in overall activity.

### Experiment-4: Functional clocks are required during SSI for after-effects

Since mutants of core clock genes may be impaired in pathways independent of circadian clock function we decided to use LL to disrupt circadian clocks in wild type *CS* flies only for the period of SSI. The activity profile, cumulative daytime and nighttime activity of SSI males do not differ from control males (Fig. 3c) indicating that functioning clocks are required in males during SSI for its after-effects to be seen later on.

Three-way ANOVA on the activity data showed a statistically significant effect of sex (*F*_1,2928_ = 81.09, *p*<0.0001), SSI (*F*_1,2928_ = 86.54, *p*<0.0001), time point (*F*_23,2928_ = 89.05, *p*<0.0001), sex×SSI (*F*_1,2928_ = 196.84, *p*<0.0001), sex×time point (*F*_23,2928_ = 17.14, *p*<0.0001), SSI×time point (*F*_23,2928_ = 1.55, *p*<0.04) and sex×SSI×time point interaction (*F*_23,2928_ = 3.17, *p*<0.0001). Post hoc multiple comparisons using Tukey's test revealed that activity of SSI males does not differ from control males at any time point tested (*p*>0.05), while in SSI females it is significantly reduced during the morning and evening activity peaks (*p*<0.01; Fig. 3c, left panels). Three-way ANOVA on the cumulative daytime and nighttime data showed a significant effect of sex (*F*_1,244_ = 11.83, *p*<0.0007), SSI (*F*_1,244_ = 12.63, *p*<0.0004), phase (day/night) (*F*_1,244_ = 105.31, *p*<0.0001), sex×SSI (*F*_1,244_ = 28.72, *p*<0.0001), sex×phase (*F*_1,244_ = 24.92, *p*<0.0001), however, the effect of SSI×phase (*F*_1,244_ = 1.35, *p* = 0.25) and sex×SSI×phase interactions are statistically not significant (*F*_1,244_ = 0.51, *p* = 0.48). Post hoc multiple comparisons using Tukey's test revealed that cumulative daytime and nighttime activity of SSI and control males does not differ (*p*>0.05), while daytime (*p*<0.0001) and nighttime activity (*p*<0.01) of SSI females is significantly reduced compared to control females (Fig. 3c, right panels), suggesting that SSI mediated after-effects are clock-independent in females.

To study the consequence of SSI on the circadian period of wild type flies, when made to interact under arrhythmicity inducing LL conditions, we recorded the locomotor activity behavior of males who had experienced 5 days of SSI under LL. The circadian period of SSI and control males does not differ (Fig. 3d). ANOVA on the circadian period data showed a significant effect of SSI (*F*_1,56_ = 0.62, *p* = 0.43). Post hoc comparison using Tukey's test revealed that circadian period of SSI and control males does not differ (*p* = 0.43) (Fig. 3d). This further confirms that functional circadian clocks in males are required during SSI for the expression of after-effects.

### Experiment-5: Males with electrically silenced LNv clock neurons show SSI mediated reduction in the evening activity peak

**Figure 4:**
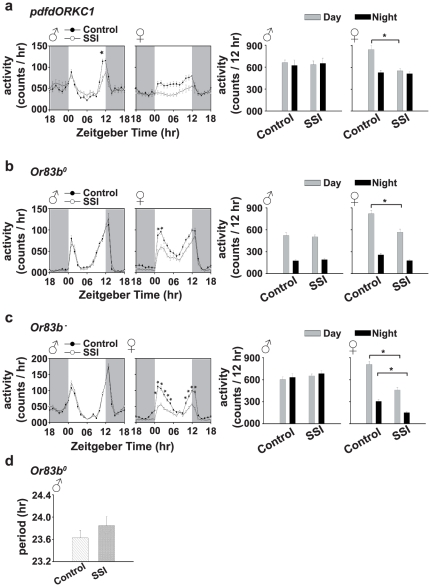
Flies with electrically silenced PDF-positive LNv clock neurons display socio-sexual interaction (SSI) mediated after-effects. (a, left panels) Activity profile of electrically silenced flies show activity of SSI males and females similar to controls except during the evening activity peak (*p*<0.05), when SSI males are less active than controls. (a, right panels) Cumulative daytime and nighttime activity of SSI control males does not differ (*p*>0.05), while in SSI females daytime activity is lower than controls (*p*<0.05) but nighttime activity is similar to controls (*p*>0.05). (b, left panels) The activity of *Or83b^0^* SSI males is similar to controls (*p*<0.05) and in females it is reduced during the morning activity peak (*p*<0.05). (b, right panels) The cumulative daytime and nighttime activity of SSI males is similar to controls, while activity of SSI females is reduced during daytime (*p*<0.05) but is similar to controls during nighttime (*p*>0.05). (c, left panels) The activity of *Or83b* ablated SSI males is similar to controls (*p*>0.05), while that of females is reduced during morning and evening activity peaks (*p*<0.05). (c, right panels) The cumulative daytime and nighttime activity of *Or83b^−^* males is similar to controls, while that of females is lower than controls (*p*<0.05). (d) Circadian period of *Or83b^0^* SSI males does not differ from controls (*p*>0.05). All other details same as in Fig. 1.

In LNv-silenced flies, SSI males show significantly reduced evening activity peak compared to control males (Fig. 4a, left panels). Three-way ANOVA on the activity data showed a statistically significant effect of sex (*F*_1,2975_ = 4.5, *p*<0.03), SSI (*F*_1,2975_ = 37.6, *p*<0.0001), time point (*F*_23,2975_ = 22.1, *p*<0.0001), sex×SSI (*F*_1,2975_ = 36.69, *p*<0.0001), sex×time point (*F*_23,2975_ = 9.59, *p*<0.0001), SSI×time point (*F*_23,2975_ = 3.6, *p*<0.0001) and sex×SSI×time point interaction (*F*_23,2975_ = 2.23, *p*<0.0006). Post hoc multiple comparisons using Tukey's test revealed that evening activity peak of SSI males is significantly reduced compared to control males (*p*<0.0001), while it does not differ from controls at any other time points (*p*>0.05; Fig. 4a, left panel). Activity of SSI females does not differ from controls at any time points tested (*p*>0.05; Fig. 4a, left panel).

Three-way ANOVA on the cumulative daytime and nighttime data showed a significant effect of SSI (*F*_1,202_ = 4.58, *p*<0.03), phase (day/night) (*F*_1,202_ = 6.62, *p*<0.01), sex×SSI (*F*_1,202_ = 4.55, *p*<0.03), sex×phase (*F*_1,202_ = 5.30, *p*<0.02), SSI×phase interactions (*F*_1,202_ = 5.17, *p*<0.02), however, the effect of sex (*F*_1,202_ = 0.68, *p* = 0.4) and sex×SSI×phase interactions are statistically not significant (*F*_1,202_ = 2.37, *p* = 0.12). Post hoc multiple comparisons using Tukey's test revealed that cumulative daytime and nighttime activity of SSI males does not differ from control males (*p*>0.05), while SSI females show significant reduction in their daytime activity compared to control females (*p*<0.0001; Fig. 4a, right panels). This suggests that LNv clock neurons in males are not required for the expression of SSI after-effects.

### Experiment-6: Functional olfactory ability in males is required during SSI for the expression of after-effects

To study the role of olfaction in the expression of SSI mediated after-effects we examined the effect of 5 days of SSI in *Or83b^0^* and *Or83b^−^* flies, known for their compromised olfactory ability (see methods). In *Or83b^0^* and *Or83b^−^* flies, activity profile, and cumulative daytime and nighttime activity of SSI males do not differ from control males (Fig. 4b, c).

Three-way ANOVA on the activity data of *Or83b^0^* flies showed a statistically significant effect of sex (*F*_1,2760_ = 94.70, *p*<0.0001), SSI (*F*_1,2760_ = 57.00, *p*<0.0001), time point (*F*_23,2760_ = 189.25, *p*<0.0001), sex×SSI (*F*_1,2760_ = 55.30, *p*<0.0001), sex×time point (*F*_23,2760_ = 14.71, *p*<0.0001), SSI×time point (*F*_23,2760_ = 1.82, *p*<0.01) and sex×SSI×time point interaction (*F*_23,2760_ = 1.59, *p*<0.03). Post-hoc multiple comparisons using Tukey's test revealed that activity of SSI males does not differ from control males at any time point tested (*p*>0.05), while of SSI females is significantly reduced compared to controls during morning activity peak (*p*<0.05; Fig. 4b, left panels). Three-way ANOVA on the cumulative daytime and nighttime data showed a significant effect of sex (*F*_1,230_ = 24.90, *p*<0.0001), SSI (*F*_1,230_ = 14.99, *p*<0.0001), phase (day/night) (*F*_1,230_ = 349.79, *p*<0.0001), sex×SSI (*F*_1,230_ = 14.54, *p*<0.0001), sex×phase (*F*_1,230_ = 11.78, *p*<0.0007) and SSI×phase interactions (*F*_1,230_ = 5.70, *p*<0.01). However, the effect of sex×SSI×phase interactions is statistically not significant (*F*_1,230_ = 2.75, *p* = 0.1). Post hoc multiple comparisons using Tukey's test revealed that cumulative daytime and nighttime activity of SSI males and nighttime activity of males does not differ from control (*p*>0.05), while SSI females show reduction in daytime activity compared to control females (*p*<0.0001; Fig. 4b, left panels). These results suggest that functional olfactory ability in males is necessary for the expression of SSI after-effects.

Three-way ANOVA on the activity data of *Or83b^−^* flies showed a statistically significant effect of sex (*F*_1,2976_ = 426.10, *p*<0.0001), SSI (*F*_1,2976_ = 97.30, *p*<0.0001), time point (*F*_23,2976_ = 210.56, *p*<0.0001), sex×SSI (*F*_1,2976_ = 219.25, *p*<0.0001), sex×time point (*F*_23,2976_ = 53.62, *p*<0.0001), SSI×time point (*F*_23,2976_ = 4.47, *p*<0.0001) and sex×SSI×time point interaction (*F*_23,2976_ = 6.25, *p*<0.0001). Post-hoc multiple comparisons using Tukey's test revealed that activity of SSI males does not differ from control males at any time point tested (*p*>0.05), while of SSI females is significantly reduced compared to controls during morning and evening activity peaks (*p*<0.05; Fig. 4c, left panels). Three-way ANOVA on the cumulative daytime and nighttime data showed a significant effect of sex (*F*_1,248_ = 74.30, *p*<0.0001), SSI (*F*_1,248_ = 16.96, *p*<0.0001), phase (day/night) (*F*_1,248_ = 59.05, *p*<0.0001), sex×SSI (*F*_1,248_ = 38.22, *p*<0.0001), sex×phase (*F*_1,248_ = 80.38, *p*<0.0001) and SSI×phase interactions (*F*_1,248_ = 4.12, *p*<0.04), however, the effect of sex×SSI×phase interaction is statistically not significant (*F*_1,248_ = 3.95, *p*<0.05). Post hoc multiple comparisons using Tukey's test revealed that cumulative daytime and nighttime activity of SSI males does not differ from control males (*p*>0.05), while SSI females show reduction in daytime (*p*<0.0001) and night time activity compared to control females (*p*<0.0001; Fig. 4c, right panels). These results further confirm that functional olfactory ability in males is necessary during SSI for the expression of its after-effects.

To study the consequence of SSI on circadian period in flies with compromised olfactory ability, we recorded locomotor activity behavior of SSI and control males under DD. Circadian period of SSI males does not differ from control males (Fig. 4d). ANOVA on the circadian period data showed a significant effect of SSI (*F*_1,34_ = 1.06, *p* = 0.31). Post hoc comparisons using Tukey's test revealed that circadian period of SSI males does not differ statistically from control males (*p* = 0.31; Fig. 4d). This suggests that olfactory ability in males during SSI is necessary for long-lasting after-effects in circadian clocks.

## Discussion

Male fruit flies *D. melanogaster* display long-lasting after-effects of SSI in their circadian activity rhythm under LD and DD conditions. The evening activity peak of SSI males is reduced and their circadian period of activity rhythm lengthened compared to control males (Figs. 1, S2). Significant changes in circadian activity rhythm can be seen in males that had experienced SSI for 4 days or more, which suggests that SSI has after-effects on circadian activity rhythm, which requires as few as 4 days of interaction between males and females to have a measurable impact (Fig. 1b). The extent of these after-effects does not change beyond 4 days of SSI and are stable over a fairly long period of time. SSI mediated after-effects require the presence of functional circadian clocks during SSI because males with loss-of-function mutation in two core clock genes (*per^0^* and *cyc^0^*) or wild type males when made to interact with females under arrhythmicity inducing LL condition [21] do not show them (Fig. 3). However, electrically silenced LNv males show SSI mediated reduction in the evening activity peak quite similar to wild type *CS* flies, suggesting that LNv clock neurons are dispensable for the expression of SSI mediated after-effects, at least under the LD conditions (Fig. 4a). Although the results of our study suggest a role for circadian clocks in the regulation of SSI mediated after-effects, in females a general decrease in activity seems to be controlled by non-circadian factors. Functional clocks are necessary during SSI because wild type males when made to interact socio-sexually with females under LL conditions do not show reduction in evening activity peak and lengthening of circadian period (Fig. 3c, d). Social communication during SSI requires functional olfactory ability because blocking them in males either by loss-of-function mutation in the *Or83b* gene or by ablating *Or83b* neurons abolishes SSI mediated after-effects on circadian activity rhythm. These results thus suggest that manipulation of circadian clocks and olfactory ability affects the expression of SSI mediated after-effects in males, while it does not cause any meaningful change in the SSI mediated after-effects in females. SSI mediated after-effects seem to be limited to males, a finding which is consistent with that of Fujii et al [12] and our own previous studies [28], wherein it was shown that males dictate socio-sexual interactions (nocturnal sex drive) and therefore it should be the males who need to adjust their circadian behaviors.

Our study is unique in that groups of flies were used which reveals how SSI occurs in nature where normally flies are found in large groups and courtship is not exclusively a one-on-one affair. The results highlight features of male-female socio-sexual communications not examined previously, and reveal that after affects of SSI modulate male and female activity rhythms in quite different ways. The results also suggest that some aspects of SSI remain long after the interactions are over, prominent being the reduction in the evening activity peak and lengthening of circadian period in males (Figs. 1, S2). Reduced evening activity peak in SSI males is reminiscent of nocturnal sex drive in which flies show increase in activity during night which continued up to the late hours of the day but a decrease in evening activity [12], [28]. Our findings are not entirely in consensus with the findings of the Fujii et al study [12], where changes such as nighttime activity and reduction in evening activity peak seen during SSI were found to revert back as soon as males and females were separated from each other. However, our study cannot be compared directly with those by Fujii et al [12], since we measured activity of individual males and females after the SSI was over rather than of groups of flies during SSI. Furthermore, we find that increased nighttime activity of males, which is considered as a marker of nocturnal sex drive or SSI returns to levels similar to controls and only the reduction in the evening activity peak persists after SSI.

The role of circadian clocks in the regulation of SSI is clear from the behavior of *per^0^* and *cyc^0^* males, who show no difference in post interaction activity between SSI and control males. To further resolve whether circadian clocks are required during SSI for the expression of after-effects later on, wild type males and females were co-housed under LL wherein locomotor activity rhythm is known to be abolished [21]. The results suggest that functional circadian clocks are critical during SSI between males and females for a long-lasting impact on circadian clocks. Previous studies have shown that nocturnal sex drive or SSI is rhythmic in *Drosophila* wherein phase of activity rhythm is shifted towards nighttime [12], [28]. Therefore, absence of SSI mediated after-effects in clock manipulated flies appears to be due to the loss of rhythmicity in SSI.

Fruit flies *D. melanogaster* show bimodal locomotor activity pattern under LD cycles, wherein activity is confined mostly to the two twilights. The morning activity peak is believed to be governed by PDF-positive LNv neurons and the evening activity peak by PDF-negative 5^th^ sLNv and LNd neurons [26], [29]. The results of our study suggest that enhanced nighttime activity observed during SSI [12] disappears as soon as males are separated from females, but reduced evening peak persists in males as a signature of after-effects of SSI (Figs. 1a, b, S2), suggesting that LNv clock neurons, known to be critical for maintaining circadian rhythmicity in activity/rest behavior under DD [26], [29] are dispensable for SSI mediated after-effects in males, at least for the LD phenotype. This view is further corroborated by the finding that evening activity peak of LNv-manipulated controls is phase advanced [24], [30], while SSI males show reduced evening activity peak (Fig. 4a). Further support for our results comes from recent studies [28], [31], wherein it was demonstrated that LNv clock neurons are dispensable for male driven sex drive.

Social communications during SSI in *Drosophila* have been proposed to occur via auditory, olfactory and mechano-sensory modalities [12]. Our study shows that olfaction plays a vital role in SSI, which is consistent with the findings of previous studies which have also implicated the role of olfaction in mediating social signals during social interactions particularly those that are sexual in nature [11]–[15], [28]. However, we do not know yet whether reduction of the evening activity peak in SSI males is merely a residual effect of SSI, or whether it has an adaptive significance for males to be less active during peak mating time. One can speculate that decrease in overall activity is likely to help females, for whom post-mating survival is more crucial, in escaping predators. In contrast increased nighttime activity would be advantageous for males as it may provide them with more opportunities to court females. Similar daytime activity in SSI exposed flies would ensure that sufficient food is acquired as amount of food acquired is known to be positively correlated to the number of eggs laid [32]. Thus, changes in activity of males and females are likely to help them adapt to challenging post mating situation in different ways.

In wild type *CS* flies, activity in SSI females is significantly reduced during the nighttime, and there is consistent decrease in daytime activity as well. As a result, cumulative activity during daytime and nighttime is reduced compared to controls (Fig. 1). This decrease in activity is seen, to a similar extent, even in the clock manipulated flies suggesting that SSI mediated after-effects in females are driven by non-circadian factors, which may be triggered by the accessory gland proteins transferred by males during mating into the reproductive tract of females, which may result in tuning down of female's physiology and behavior [33]–[35]. When SSI is allowed to occur under LL, the outcome remains more or less similar, further confirming the role of non-circadian factors in the regulation of post mating reduction of activity levels in females [33], [34]. In LNv-silenced flies, nighttime activity of SSI females is comparable to controls; however, daytime activity is reduced, which suggests that LNv clock neurons may have some role in keeping the nighttime activity in SSI females to levels lower than controls. Blocking of olfactory responses does not alter nighttime activity of SSI females, while their daytime activity is reduced. This may be due to the fact that olfactory responses peak during night [36] and therefore social cues may be more effective during nighttime. These results suggest that SSI mediated after-effects in females are non-circadian in nature.

Is SSI mediated after-effects on circadian clocks a real phenomenon or simply a random variation? In *Drosophila*, it is well known that social enrichment (experience) can alter many aspects of the fly behaviors including its circadian rhythm [11]–[16], [28]. Social experience is a key determinant of mating frequency of the group and is known to alter expression of several clock genes in the brain and other peripheral organs [13]. In *Drosophila*, after social enrichment there is increase in sleep. Further such experience of social enrichment is associated with an increase in the number of synaptic terminals in the LNv projections into the medulla [37]. Social enrichment is also known to cause long lasting neuroanatomical changes in the fly [37]–[41]. Based on the above findings it would be reasonable to assume that socio-sexual interactions between males and females could cause some impact on the circadian timing system of *Drosophila*. Taken together the results of our studies, supported by statistical analysis, of data collected in separate sets of experiments, done at different points of time, confirms that the SSI mediated after-effects on circadian clocks is real. However, further studies from other groups on separate wild type strains of *Drosophila* would be required to confirm the universality of this phenomenon.

In summary, SSI in fruit flies *D. melanogaster* results in long-lasting changes in circadian clocks. In males, it results in reduction of evening activity peak and lengthening of circadian period, while in females it causes a reduction in overall activity. This was further confirmed in a separate set of experiments confirming that SSI mediated behavior is reproducible. Functional clocks are necessary during SSI for its rhythmic regulation because males with permanently or temporarily (only during SSI) compromised circadian clocks do not display such after-effects. To the best of our knowledge this is the first report of its kind demonstrating consequence of SSI on circadian clocks in fruit flies *D. melanogaster*, and the role of functional clocks and olfaction in SSI mediated after-effects.

## Supporting Information

Figure S1 **Protocol for experiment 1A and 1B. Experiment-1A:** Virgin *CS* males and females were maintained up to 4 days of age as same sex groups of 30 individuals per vial. After 4 days, flies were segregated into five sets, of which the first was maintained as same sex groups - males (30 ♂) or females (30 ♀) (0 days of socio-sexual interaction - SSI) until age day 10. Remaining four sets comprised males and females that were mixed in 1∶1 ratio (15 ♂+15 ♀ per vial) on different days, which enabled SSI to take place at different stages. The schematic shows the age of flies in the extreme left column followed by the control (0 days group). Columns under SSI show the schedule of mixing in each of the experimental groups. Solid arrows indicate flies kept as same-sex groups while hollow arrows indicate mixed (SSI) status. Males and females of one set were mixed on the 5^th^ day and kept together for next five days (5 days of SSI), another set were mixed on the 6^th^ day and kept together for next four days (4 days of SSI), next set were mixed on the 7^th^ day and kept together for next three days (3 days of SSI) and the last set were mixed on the 8^th^ day and kept together for next two days (2 days of SSI), such that SSI in all groups ended on the same day. Flies (SSI and control) were transferred into fresh food vials every day until termination of SSI. On completion of SSI (age day 10), males from both control and SSI groups were separated and introduced individually into glass tubes to monitor their locomotor activity behavior under light/dark (LD) cycles for at least 7 days. **Experiment-1B:** Protocol was similar to experiment 1A except for the following. Controls (0 days socio-sexual interaction - SSI) remained in same-sex groups till age day14. Males and females of one of the experimental sets were mixed on the 5^th^ day and kept together for next ten days (10 days of SSI), of another set on the 7^th^ day and kept together for next eight days (8 days of SSI) and last set mixed on the 10^th^ day and kept together for next five days (5 days of SSI). On completion of SSI (age day 14) activity of individual males were assayed under light/dark (LD) cycles for at least 7 days.
(EPS)

Figure S2 **Socio-sexual interactions (SSI) reduce evening activity peak and are stable in flies interacting for 5 days or more.** Activity profiles of males following 5, 8 or 10 days of SSI. Zeitgeber Time (ZT in hours) is plotted along the x-axis, while activity counts in one hour bins averaged across five days is plotted along y-axis. The shaded area indicates night and the empty area day of laboratory light/dark (LD) cycles. The error bars are standard error around the mean (SEM). Asterisks indicate significant differences with *p* values<0.05. The evening activity peak (activity in 1 hr bin between ZT12 and ZT13) of males exposed to SSI for 5 or 8 or 10 days is significantly reduced compared to control males (*p*<0.0001). At all other time points, activity of SSI and control males does not differ (*p*>0.05). Although there is an appreciable reduction in the morning activity peak of SSI males, this change did not reach statistical levels of significance (*p*<0.05). This suggests that 5 days of SSI is effective in causing long-lasting changes in the evening activity peak of males as 8 or 10 days of SSI.
(EPS)
